# Interferons in human inborn errors of disease

**DOI:** 10.1128/mbio.01570-25

**Published:** 2025-06-24

**Authors:** Laurel Stine, Sara Cahill, Fiachra Humphries

**Affiliations:** 1 Division of Innate Immunity, Department of Medicine, University of Massachusetts Chan Medical School 164186, Worcester, Massachusetts, USA; The Ohio State University, Columbus, Ohio, USA

**Keywords:** innate immunity, interferons, viruses, TLR signaling, cGAS STING

## Abstract

Interferons are ubiquitously produced cytokines with diverse cellular functions. IFNs play essential functions in responding to viral infections and tumorigenesis by initiating an interferon-stimulated gene response and triggering the adaptive immune response. However, excessive, prolonged IFN production disrupts cellular homeostasis and can lead to an array of severe inflammatory conditions. Due to developments in whole genome sequencing and elucidation of key signaling pathways, excessive IFN production and its associated interferon-stimulated gene expression signature is now an established molecular diagnostic of Mendelian inborn errors in immunity termed “interferonopathies.” In addition, identification of Mendelian loss-of-function mutations in critical mediators of the IFN response has been identified as the causal factors of immune deficiencies and susceptibility to viral infections. Thus, IFNs and their preceding signaling pathways now represent major therapeutic targets. In this review, we outline the existing evidence on the role IFNs play in human disease and the genetic mechanisms that underlie excessive IFN production or immune deficiencies.

## INTRODUCTION

Interferons (IFNs) are a family of pleiotropic cytokines traditionally associated with anti-viral and anti-tumor immunities (1). IFNs are ubiquitously produced in response to infection, damage, or cellular stress, following the detection of pattern-associated molecular patterns (PAMPs) by pathogen-recognition receptors (PRRs), such as toll-like receptors (TLRs) and nucleic acid sensors (2, 3). IFNs are categorized into three distinct subsets: type I, type II, and type III (4). Type I IFN can be ubiquitously expressed by many cell types, and type II IFN is typically produced by immune cells. Type III IFNs, which distinctly signal through the type III receptor, are produced at mucosal surfaces such as respiratory epithelial cells and intestinal epithelial cells. Although all subgroups display distinct functions and tissue-specific expression, their downstream signaling cascades are mediated through a class of adaptor proteins and transcription factors known as Janus kinase (JAK)-signal transducer and activator of transcription (STAT), which trigger the transcription of IFN-stimulated genes (ISGs). ISGs are a diverse set of proteins that can restrict viral replication by directly targeting numerous elements of viral entry and replication mechanisms (5). Additionally, IFNs can stimulate the activation of CD8 T cells, regulate APC function, stimulate antibody production from B cells, and induce the production of chemokines that can promote the recruitment of immune cells and tissue damage (6, 7). Although IFNs play important roles in host immunity, their potent nature requires significant regulation by several checkpoint mechanisms to limit pathogenic effects (8, 9).

Many monogenic autoinflammatory diseases have been attributed to single-gene mutations in innate immune-signaling pathways that present early in life as sterile inflammation, mirroring symptoms of infection (10). Excessive IFN production and associated ISG signatures have been identified as important indicators of monogenic immune-mediated diseases termed “interferonpathies” (10, 11). Interferonopathies are a form of inherited autoinflammatory disease characterized by constitutive activation of the IFNAR pathway (10, 12). Due to the recent advances in genome sequencing, the majority of interferonopathies have been attributed to the loss-of-function (LOF) or gain-of-function (GOF) monogenic mutations in proteins that regulate the detection of nucleic acids or IFNAR signaling (13–20). Despite common underlying signaling defects, specific in-born errors can lead to an array of clinical manifestations. In addition, the sporadic, periodic nature of some interferonopathies creates challenges for treatment. Thus, further understanding of the molecular and cell-specific signaling events that precede IFN production and the ISG signature is essential for differentiating DNA- and RNA-driven disease and developing more targeted precision therapies.

In this review, we discuss the key innate immune receptors and signaling events that precede IFN production, the underlying genetic causes of excessive IFN production, and the ISG signature as a danger signal in human disease. We also discuss the existing evidence for ISG signatures in other non-Mendelian autoimmune diseases and explore the danger signals that precede excessive ISG activation. Finally, we describe the signaling events and hierarchy of clinical symptoms within a group of immunodeficiencies resulting from LOF mutations PRR pathways.

## PRODUCTION OF IFN

### Toll-like receptors

TLRs are a conserved family of PPRs that induces the production of IFNs following the detection of PAMPs (2). Plasma membrane and endosomal TLRs signal via the TIR adaptors Myd88, TRIF, and TRAM to induce type I IFN in response to infection (21). TLR-4 is a lipopolysaccharide (LPS) sensor localized to the plasma membrane. Following the detection of LPS, TLR-4 is endocytosed via its interaction with the adaptor proteins TRIF and TRAM (22). TRIF and TRAM recruit the E3-ligase TRAF3 to facilitate TBK1 activation and IRF3 phosphorylation (23). Phosphorylated dimeric IRF3 then translocates to the nucleus and associates with IRF-binding elements in the promoter of anti-viral genes, including IFNβ (24). Expression of IFNβ occurs following assembly of the IFNβ enhanceosome. A heterodimer of NF-κB p50 and p65 and a heterodimer of the basic region-leucine zipper (bZIP) proteins ATF-2 and c-jun bind to PRDII and PRDIV, respectively. PRDIII and PRDI are recognized by a protein complex containing IFN regulatory factor 3 (IRF-3) (25).

**Fig 1:**
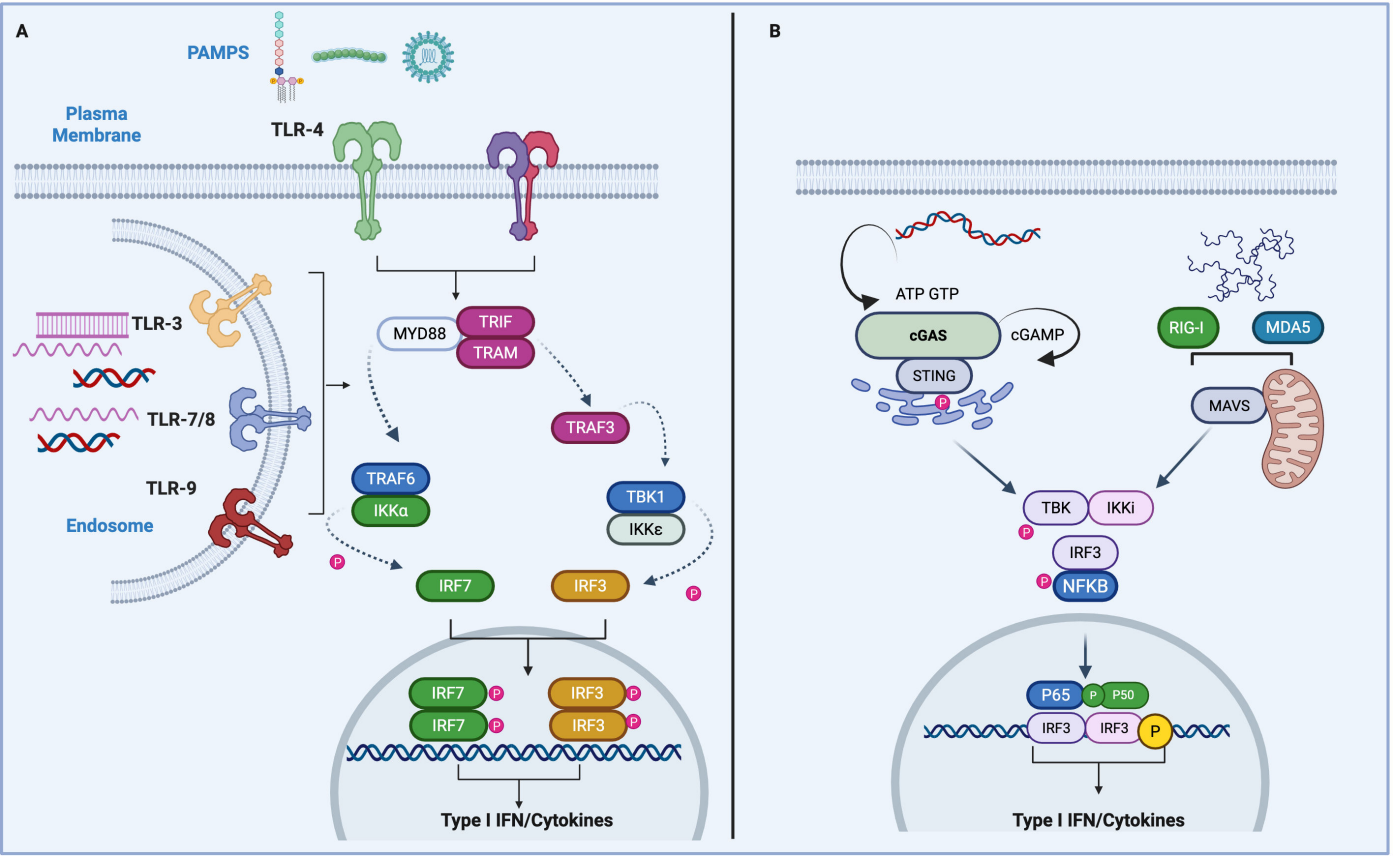
(**A**) TLR Signaling and IRF transcription factors promote IFN production. TLRs located at the plasma membrane or endosomes detect conserved microbial patterns, leading to the recruitment of TIR-containing adaptor proteins and the activation of Myd88-dependent or -independent signaling cascades that result in IFN expression via the transcription factors IRF3 and IRF7. Recruitment of Myd88 triggers the activation of TRAF6 and the IKK complex, which facilitates IRF7 activation and expression of IFN-α. TRIF and TRAM recruitment induces the auto-ubiquitination of TRAF3 and activation of the kinases TBK1 and IKKε. TBK1 and IKKε phosphorylate IRF3, which translocates and induces the expression of type I IFN-β. Signaling via TLR3 occurs in a Myd88-independent manner via the adaptors TRIF, TRAF3, TBK1, and IKKε. (**B**) DNA- and RNA-sensing pathways and type I IFN production. Schematic representation detailing the detection and signaling mediated via nucleic acid sensors. Detection of dsDNA results in a conformational change and the activation of cGAS enzymatic activity. cGAS synthesizes cGAMP from the precursors ATP and GTP. cGAMP binds to STING dimers at the ER membrane, which triggers conformational changes to facilitate STING oligomerization and translocation to the ER–Golgi intermediate compartment (ERGIC) and Golgi, where STING recruits TBK1. STING-mediated TBK1 recruitment leads to TBK1 autophosphorylation, STING phosphorylation recruitment of IRF3. Phosphorylated IRF3 can then dimerize and translocate to the nucleus to induce the expression of type I interferons. In addition to IRF3, STING also promotes NF-κB activity and the formation of LC3-bound autophagosomes through a non-canonical autophagy pathway. RIG-I and MDA5 can sense immunostimulatory RNA that enters the cytosol, from viral or endogenous sources. Following the detection of RNA, RIG-I or Mda5 undergoes conformational changes to allow exposure and multimerization of CARD domains. CARD–CARD multimers form interactions with the downstream adaptor protein MAVS. MAVS is localized to the outer membranes of mitochondria and peroxisomes. Following activation, MAVS forms prion-like oligomers, which facilitate the activation of TBK1 IKKε, which can activate IRF3 and NF-κB to induce the expression of type I IFNs and proinflammatory cytokines.

Endosomal TLRs (TLR3,7, 8, and 9) detect nucleic acids, including DNA and RNA, enabling a broad-spectrum immune response to a wide range of pathogenic bacteria and viruses (26). Endosomal TLRs are synthesized in the ER and transported to endosomes via the chaperone protein UNC93B1. UNC931B is required for correct folding of endosomal TLRs and ligand recognition. TLR-7 remains bound to UNC93B1 at the endosome in association with syntenin-1 to internalize TLR-7 in micro-vesicles and restrict TLR-7 activation by single-stranded RNA (ssRNA) (27). TLR-8 is primarily activated by RNA degradation products, specifically catabolic uridine and purine-2′,3′-cyclophosphate-terminated oligoribonucleotides, generated by the lysosomal endoribonuclease RNase T2 (28). In contrast, TLR-9 is regulated differently and is released by UNC93B1 in order to bind CpG DNA and trigger downstream signaling (26). These unique trafficking mechanisms play important roles in limiting TLR-7- and 9-dependent autoimmune conditions (6, 29). UNC93B1 deficiency results in impaired TLR-3, 7, and 9 responses and hyper-susceptibility to viral infections (30). In pDCs, activation of TLR-7 and TLR-9 induce IFN-α expression via the transcription factor IRF7. Following activation, TLR-7 and TLR-9 recruit a complex comprised of MyD88 IRAK1/4 and the E3 ligase TRAF6 (31, 32). TRAF6-mediated activation of IKK-α leads to the phosphorylation and translocation of IRF7 to initiate IFNα expression (33) (Fig. 1A).

Unlike all other TLRs, TLR3, which is activated by double-stranded RNA, signals in a Myd88-independent manner via the adaptor protein TRIF. Downstream of TLR-3, TRIF recruits the E3 ligase TRAF3 and initiates the activation of TBK1- and IRF3-induced IFN expression (23, 34). Endosomal TLRs are essential mediators of human anti-viral immunity. Indeed, loss-of-function mutations in components of the endosomal TLR-signaling pathway result in susceptibility to herpes simplex virus infection (HSV1) (6, 30, 35–37). In contrast, endosomal TLRs have also been implicated in the pathogenesis of autoimmune conditions such as SLE (29, 38, 39)

## NUCLEIC ACID SENSORS

### cGAS/STING signaling

Cyclic GMP-AMP (cGAMP) synthase, cGAS, is an innate immune sensor of cytosolic DNA (40). Binding of cGAS to pathogen- or host-derived dsDNA promotes a conformational change activating cGAS catalytic activity and the conversion of ATP and GTP into the non-canonical cyclic dinucleotide cGAMP (40, 41). cGAMP then binds and activates the stimulator of interferon genes (STING) (3, 40). Following cGAMP binding, TBK1 is recruited to the C-terminal tail of STING, which induces auto-activation and phosphorylation of TBK1, followed by phosphorylation of STING on Ser366 (42). STING can also be activated directly via bacterial-derived cyclic dinucleotides (43). Conformational changes in STING promote its oligomerization and induction of downstream-signaling pathways, such as type I IFN, NFκB, and autophagy (34, 44–47). Upon activation, STING is trafficked from the ER to the Golgi and finally to the lysosome for degradation. Given the damaging consequences of DNA accumulation, several regulatory mechanisms exist to restrict spontaneous cGAS STING signaling. Under resting conditions, STING translocation is restricted by the ER protein STIM1 and by COPI vesicles via the adaptor protein SURF4 (48, 49). Autophagy can also be activated downstream of STING, which results in the phosphorylation of STING by ULK1 to limit pathway activation (50) In addition, several regulatory mechanisms restrict cGAS binding to host DNA. cGAS is localized to both the cytosol and nuclear compartment in close proximity to genomic DNA (51). In the nucleus, cGAS is tightly bound to chromatin and locked in an inactive state whereby steric hindrance prevents the association of its DNA binding domain with genomic DNA (51–55). Disruption of STING trafficking and cGAS localization underpin the mechanisms of such as COPA syndrome, SAVI, and certain forms of AGS (56–58) (Fig. 1B). In addition to cGAS and STING, other dsDNA sensors play important roles in responding to pathogens. The STING-independent DNA-sensing pathway (SIDSP) is mediated by DNA-PK in human cells to initiate a broad anti-viral response. (59)

### RIG-I/Mda5 signaling

RIG-I-like receptors (RLRs) receptors are cytosolic sensors of viral RNA. The RLR family of sensors is comprised of RIG-I, Mda5, and LGP2 (60). RLRs sense unique viral RNA products from viral species such as picornaviruses, flaviviruses, and paramyxoviruses (61). Furthermore, RLRs are activated by host-derived RNAs (62). Upon detection of viral RNA, the RLRs recruit the adaptor protein mitochondrial antiviral signaling (MAVS) (63). Both RIG-I and Mda5 contain an N-terminal caspase recruitment domain (CARD), which facilitates the recruitment of MAVS. Although RIG-I and Mda5 have conserved homology, the specific RNA types and structures they detect are different. RIG-I specifically detects RNA molecules bearing a triphosphate (PPP) group at their 5′ end (64). 5′-PPP RNA structures are found in viral RNA molecules but not in the majority of host cytosolic RNAs, enabling RIG-I to be highly selective in detecting pathogen RNA. In contrast to RIG-I, Mda5 can be activated by viral, host, and synthetic RNA with length and secondary structure of the RNA being essential factors in Mda5 RNA sensing (41). Indeed, Mda5 is activated by higher-order dsRNA molecules (65). LGP2 was originally identified as a negative regulator of RIG-I and Mda5 signaling due to the lack of CARD domain and its ability to bind viral RNA with high affinity. Subsequent studies identified a key role for LGP2 in mediating RIG and Mda5 signaling (60). Ubiquitin chains play important roles in both TLR and Mda5 activation. K63-linked ubiquitination of RIG-I by TRIM25 is essential for the activation and recruitment of MAVS (66). RIG-I is also negatively regulated by the E3 ligases Cbl, RNF122, and RNF125 via K48-linked ubiquitination (67, 68). Linear ubiquitination by the LUBAC complex has been shown to destabilize the RIG-I TRIM25 interaction to suppress activation (69). Mda5 ubiquitination by TRIM65 is a prerequisite for Mda5 oligomerization. In addition, activation of RIG-I and Mda5 can also be achieved via unanchored ubiquitin chains (70). Deubiquitination is also a key step in regulating the RIG-I pathway. Deubiquitinating enzymes such as CYLD, USP3, and USP21 impair RIG-I activity via the removal of K63-linked chains (60). Oligomerization of MAVS into prion aggregate-like structures facilitates the recruitment of TRAF2, 5, and 6 and the downstream activation of the kinases TBK1 and IKKε (71, 72). TBK1 and IKKe phosphorylate and activate IRF3 and IRF7 to induce type I IFN and ISG expression. Both RIG-I and Mda5 activities are restricted by basal phosphorylation events (60). Phosphorylation of RIG-I impairs auto activity by repressing its interaction with TRIM25. Additionally, dephosphorylation is a key step in Mda5 activation (70).

Mda5 plays an important role in limiting RNA virus infection, and a large body of genetic evidence has highlighted the importance of Mda5 in the context of disease and anti-viral immunity. Inborn errors in IFIH1, the gene encoding Mda5, have also been attributed to the development of interferonopathies and other autoimmune conditions mediated by IFNs. Indeed, GOF function mutations in Mda5 result in constitutive IFN signaling and the development of AGS (17, 19, 73). Furthermore, the Mda5 A946T allele has been identified as a risk factor in the development of a number of autoimmune diseases including type 1 diabetes, SLE, and multiple sclerosis (74). Loss-of-function mutations in Mda5 have also been identified in children with severe respiratory viral infections (75). In addition to RIG-I and Mda5, dsRNA sensors PKR and OAS also respond to viral infections (76–78).

## INTERFERON SIGNALING

### Type I interferon

**Fig 2:**
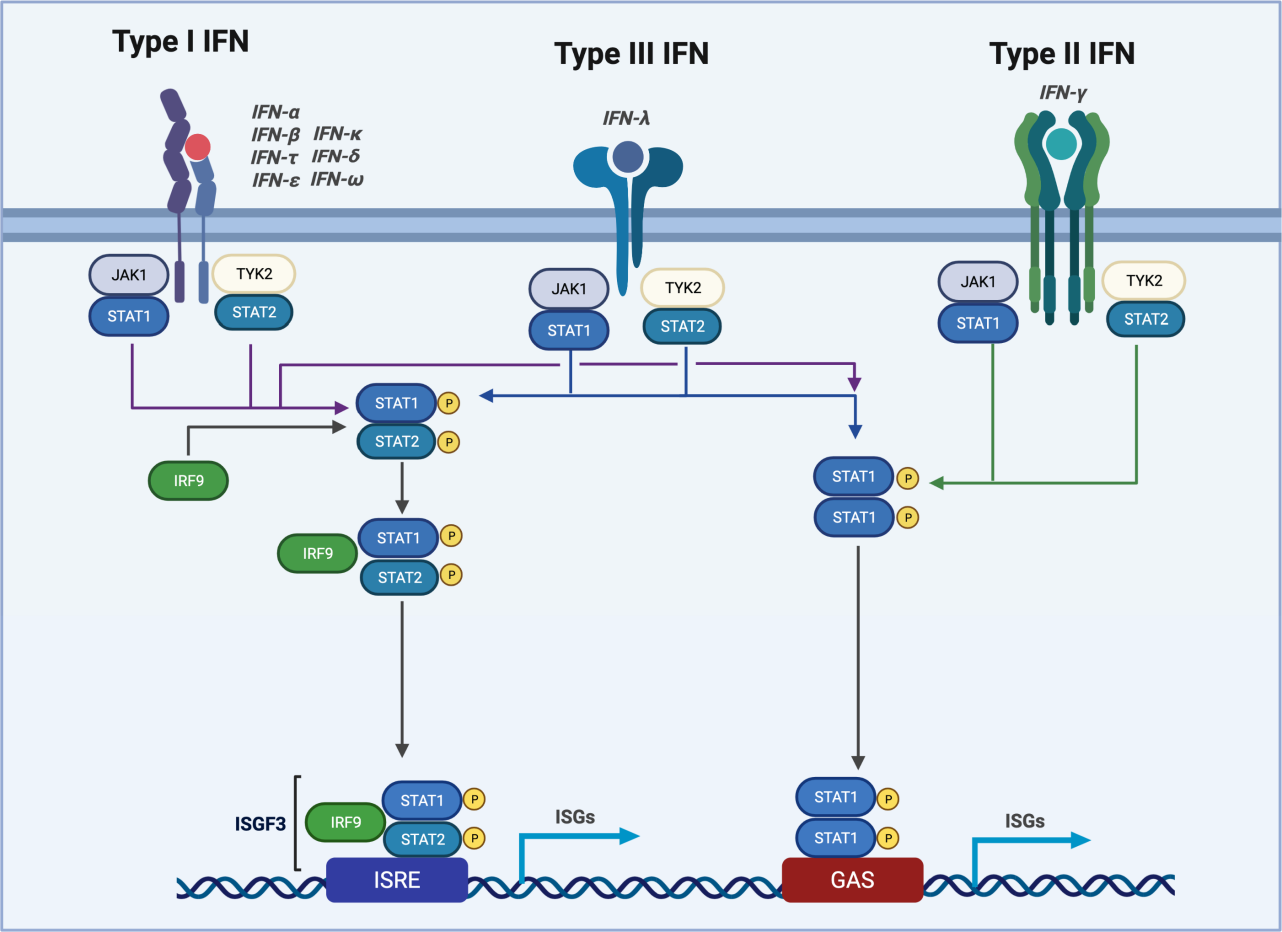
Type 1, type II, and type III IFN signaling. Schematic representation of type I-III IFN receptor activation and the resulting signaling cascades. Type I, III, and type II IFNs trigger activation of both JAK1 and the TYK2 adaptor protein, which facilitates STAT phosphorylation. Phosphorylated STAT1 can then form heterodimers with STAT2 to recruit IRF9. STAT1, STAT2, and IRF9 then form the interferon-stimulated gene factor 3 (ISGF3) transcription factor complex. ISGF3 translocates to the nucleus and binds the interferon-stimulated regulatory element (ISRE) sequences for induction of ISG expression.

Production of type I IFN is an essential early component of the anti-viral immune response and is ubiquitously produced by many cell types. Type I IFNs include IFN-α isoforms along with IFN-β, IFN-ε, IFN-κ, IFN-ω, IFN-δ, IFN-ζ, and IFN-τ. Human IFNβ and α monomers bind to the ubiquitously expressed heterodimer IFNAR1 and IFNAR2 receptors. Ligation of IFNs to their cognate receptors mediates high affinity binding and signal transduction via the kinases TYK2 via IFNAR1 and JAK1 via IFNAR2, resulting in phosphorylation of IFNAR1, which enables the recruitment of STAT2, followed by phosphorylation of STAT2 and recruitment of STAT1 (79). STAT1 is a latent cytoplasmic transcription factor, which becomes activated in response to type I, II, and III IFNs. STAT1 STAT2 heterodimers can then translocate to the nucleus in tandem with the transcription factor IRF9 to form an interferon-stimulated gene factor 3 heterotrimer (ISGF3). ISGF3 binds to interferon-stimulated response elements (ISRE) in the promoters of target genes via the DNA-binding domains of STAT1 and IRF9 (80). IFN signaling can also induce the expression of IRF7, which facilitates a positive feed-forward loop to further enhance IFN- α production (81) (Fig. 2).

### Type II interferon

Type II IFN, IFNγ, plays critical roles in anti-viral and anti-bacterial immunity. IFNγ is primarily produced by natural killer cells during infection. Like type I IFNs, IFNγ promotes an anti-viral response via the induction of ISGs. Type I IFN, IL-12, IL-15, and IL-18 stimulation of NK results in the expression of IFNγ (82). IL-18 can induce the production of IFNγ via the transcription factors STAT1 or STAT4 (83). IFNγ binds the IFNγ receptors IFNGR1 and IFNGR2 and signals downstream to activate JAK1 and JAK2 (84). IFNγ plays broader anti-viral roles by enhancing antigen presentation to T cells, stimulating nitric oxide production to inhibit viral replication, and promoting vasodilation (85). IFNγ can also promote M1 macrophage polarization for efficient production of IL-12, TNF- α, and IL-1β (86) (Fig. 2).

### Type III interferon

Like type I and type II, type III IFNs play important roles in anti-viral immunity. Type III IFNs consist of IFNλ subtypes 1-4 in humans. IFNλ signal through a dimeric receptor consisting of the IFNLR1 and the β-subunit of the IL-10 receptor (87). Expression of IFNλ is limited to epithelial cells, B cells, and neutrophils. Thus, IFNλ plays an important role in restricting viral replication at mucosal surfaces and barrier sites. IFNλ also signals via the JAK-STAT signaling pathway to induce an ISG response. However, type III signaling is regulated differently and provides a more sustained ISG response in epithelia when compared with other cell types (88, 89) (Fig. 2).

## NUCLEIC ACID-ASSOCIATED INTERFERONOPATHIES

### Aicardi Goutières syndrome (AGS)

AGS is a genetically heterogeneous disease primarily affecting the skin and the brain. AGS is clinically defined as a viral encephalitis-like syndrome, which leads to demyelination, basal ganglia calcifications of the CNS, and developmental delay (90). In all cases, AGS is characterized by excessive production of type I IFN. The AGS-associated ISG molecular signature in patient cells has become a key biomarker of AGS and an important early diagnostic tool (10, 14). To date, mutations in nine different genes have been attributed to AGS including TREX1 (13), SAMHD1 (15), ADAR1 (16), MDA5 (17), RNASEH2 subunits A, B, and C (91), LSM11, and RNU7-1 (20) (Table 1. All mutations result in constitutive IFN production and ISG expression because of aberrant accumulation and detection of immunostimulatory nucleic acids. Mutations in AGS-linked genes lead to the accumulation of host-derived DNA and RNA as a result of a breakdown of regulatory mechanisms, which normally restrict the detection of nucleic acids in the cytosol. Detection of host-derived nucleic acids in the cell leads to the persistent activation of type I IFN and its associated ISG signature via a range of nucleic acid sensors. Given the comparable symptoms with congenital infections exclusion of viral infection is first confirmed in diagnosing AGS, followed by genome sequencing to identify possible AGS-linked mutations (10). Genome sequencing of patients exhibiting the clinical symptoms of AGS has enabled the identification of new AGS mutations in previously uncharacterized cases. In recent years, the mechanistic basis of AGS has been attributed to the constitutive activation of the cGAS STING DNA-sensing pathway or the RLR receptors RIG-I and Mda5.

**TABLE 1:** Summary of conditions associated with mutations in the IFN pathway

Condition	Gene	Mutations	Mutation phenotype	Pathology	Treatment	References
Aicardi Goutières syndrome (AGS)	*TREX1*	R114H	LOF	Developmental delay, encephalopathy, skin rash	JAK inhibitors, steroids	(13, 92)
	*SAMHD1*	H123Y, R143H/C, R145Q	LOF	Developmental delay, encephalopathy, skin rash	JAK inhibitors, steroids	(15, 93)
	*ADAR1*	P193A	LOF	Developmental delay, encephalopathy, skin rash	JAK inhibitors, steroids	(16, 94, 95)
	*IFIH1*	R720H, R779H, R337G, R779H, A779C, G495R, N393V, R720G	GOF	Developmental delay, encephalopathy, skin rash	JAK inhibitors, steroids	(73, 96, 97)
	*RNASEH2*	A177T	LOF	Developmental delay, encephalopathy, skin rash	JAK inhibitors, steroids	(98–100)
	*LSM11*	G211S	LOF	Developmental delay, encephalopathy, skin rash	JAK inhibitors, steroids	(20, 101)
	*RNU7-1*		LOF	Developmental delay, encephalopathy, skin rash	JAK inhibitors, steroids	(20, 101)
Sting-associated vasculopathy with onset in infancy (SAVI)	*STING*	V155M, N154S, V147L, R281Q	GOF	Interstitial lung disease, skin lesions	Cyclophosphamide JAK inhibitors	(57, 102–107)
COPA syndrome	*STING*	P145S,K230N,R233H,W240R, E241K, E241A, D243N, A239P	LOF	Interstitial lung disease	JAK inhibitors	(48, 56)
Chronic mucocutaneous candidiasis (CMC)	*STAT1*	R274Q	GOF	*Candida* infection	Anti-fungals	(108–110)
ISG15 deficiency	*ISG15*	V104M	LOF	Cerebral calcification, skin lesions, systemic inflammation	JAK inhibitors	(8, 111–113)
USP18 deficiency	*USP18*	I60N	LOF	Cerebral calcification, skin lesions, systemic inflammation	JAK inhibitors	(113, 114)
CANDLE/PRAAS	*PSMB8*	G197E	LOF		Glucocorticoids, JAK inhibitors	(106, 115–118)
	*PSMB9*	G156D	LOF		Glucocorticoids, JAK inhibitors	(115–119)
	*PSMB10*	G201R	LOF		Glucocorticoids, JAK inhibitors	(115–119)
Otulin related autoinflammatory syndrome (ORAD)	*OTULIN*	G281R, L272P	LOF	Systemic inflammation	Prednisone, colchicine, bone marrow transplant	(120, 121)
Severe viral disease	*TLR3*	P554S	LOF	HSV1 encephalitis, severe respiratory virus infection	Acyclovir, Paxlovid, vaccination	(35)
	*TRIF*	S186L	LOF	HSV1 encephalitis, severe respiratory virus infection	Acyclovir, Paxlovid, vaccination	(37)
	*UNC93B1*	P404S	LOF	HSV1 encephalitis, severe respiratory virus infection	Acyclovir, Paxlovid, vaccination	(122, 123)
	*TRAF3*	R118W	LOF	HSV1 encephalitis, severe respiratory virus infection	Acyclovir, Paxlovid, vaccination	(23, 36)
	*TBK1*	D50A, G159A	LOF	HSV1 encephalitis, severe respiratory virus infection	Acyclovir, Paxlovid, vaccination	(122, 124)
	*IRF3*	R285Q	LOF	HSV1 encephalitis, severe respiratory virus infection	Acyclovir, Paxlovid, vaccination	(122, 125)
	*IFNAR*	1,675 bp deletion	LOF	HSV1 encephalitis, severe respiratory virus infection	Acyclovir, Paxlovid, vaccination	(122, 126)

#### DNA accumulation and cGAS/STING-mediated AGS

TREX1 is an abundant 3′-5′ exonuclease, which digests cytoplasmic DNA and prevents unwanted activation of nucleic acid sensors (92). TREX1 mutations were first identified in AGS patients presenting with severe encephalitis, intracranial calcifications, and elevated type I IFN in the CSF (13). In addition to AGS, mutations in TREX1 have also been identified in familial chilblain lupus, retinal vasculopathy, and SLE (127). Since its attribution to AGS, many experimental studies demonstrated that *Trex1* deficiency results in the abnormal accumulation of DNA species in the cytosol and the constitutive activation of cGAS and STING (13, 92, 128–131). Indeed, *Trex1*-deficient mice have reduced post-natal survival and develop severe myocarditis (128). Trex1-deficient mice exhibit systemic inflammation, production of autoantibodies to dsDNA, and renal disease (129). In contrast to human AGS mutations in *TREX1*, to date, no neurological phenotypes have been identified in *Trex1-*deficient mice.

RNase H2 enzymes are ubiquitously expressed and partner with RNase H1 enzymes to degrade cellular RNA:DNA hybrids that arise during nuclear DNA replication and R-loop formation (98). RNase H2 catalyzes ribonucleotide excision repair through the cleavage and removal of single ribonucleotides incorporated into DNA (99). LOF mutations in the RNase H2 genes *RNASEH2A*, *RNASEH2B,* and *RNASEH2C* result in a decrease in stability and reduced levels of cellular ribonucleotide excision repair. RNase H2 mutations account for half of AGS mutations. However, approximately 30% of AGS patients with *RNASEH2B* mutations do not present with a constitutively active ISG signature. Despite accounting for many AGS cases, there has been a limited number of mechanistic studies investigating how disruption of the RNase H2 complex results in AGS. An experimental knock-in mouse model incorporating a common RNase H2B mutation, A177T (RnaseH2B^A177T/A177T^) recapitulated the constitutive ISG signature observed in patients (100). Despite recapitulation of the ISG signature, *RnaseH2B^A177T/A177T^* did not develop any age-associated defects or neurological symptoms observed in AGS patients. Interestingly, *RnaseH2B^A177T/A177T^* crossed to Sting-deficient mice resulted in a significant reduction in the ISG signature when compared with *RnaseH2B^A177T/A177T^* mice (100).

SAMHD1 is a dGTP-stimulated triphosphohydrolase that converts deoxynucleoside triphosphates into deoxynucleotides and inorganic triphosphate. Since its discovery, several broad regulatory functions have been attributed to SAMHD1 dNTPase activity including restriction of viral replication, maintenance of genome stability, cell cycle progression, tumor suppression, and immune responses (15, 93, 132, 133). Mutations in SAMHD1 represent a small subset of AGS patients and impair the oligomerization and enzymatic activity of SAMHD1 (15). SAMHD1-linked AGS patients present with early onset encephalitis, intracranial calcifications, and the accumulation of IFN-α in the cerebrospinal fluid (15). SAMHD1 is highly expressed in dendritic cells and has been identified as an HIV-1 restriction factor (132). During infection, SAMHD1 prevents the reverse transcription and the synthesis of retroviral cDNA such as HIV through the hydrolysis of cellular dNTPs. Indeed, HIV replicates more readily in SAMHD1-deficient monocytes and T cells (132).

SAMHD1 is heavily regulated by post-translation modifications including phosphorylation in cycling cells via CDK1 kinase (134). Phosphorylation of SAMHD1 at T592 destabilizes the SAMHD1 tetramer and impairs its triphosphohydrolase activity in cells undergoing mitosis. SAMHD1 is dephosphorylated by PP2A in terminally differentiated cells, thus limiting its activity to non-cycling cells (135). In addition to phosphorylation, SAMHD1 is also regulated by acetylation and cysteine oxidation (133, 136). During mitosis, ARD1-mediated acetylation of K405 in SAMHD1 enhances dNTPase activity, leading to a reduction in the cellular levels of dNTPs (133). Cysteine oxidation of SAMHD1 constitutes a redox switch during proliferation, which impairs tetramerization, catalytic activity, and nuclear localization (136). SAMHD1-deficient mice display a constitutive ISG signature but do not develop any clinical symptoms of AGS (137). Although the precise nature of SAMHD1-mediated suppression of type I remains unclear, it has been proposed that SAMHD1 deficiency may permit the accumulation of retroviral nucleic acids that trigger nucleic acid sensors and the induction of IFN and ISG expressions. The constitutive expression and tight regulation of SAMHD1 by PTMs represent opportunities for direct therapeutic targeting to regulate SAMDH1 activity. Furthermore, the manipulation of SAMHD1 acetylation and cysteine oxidation may also be an alternative strategy for limiting retroviral replication.

The most recent forms of AGS have been attributed to LOF mutations in LSM11 and RNU7-1. Using exome sequencing, mutations in LSM11 and RNU7-1 were identified in AGS patients without a genetic diagnosis (20). LSM11 and RNU7-1 are components of the U7 small nuclear ribonucleoprotein (snRNP) complex, which processes replication-dependent pre-mRNAs. In the absence of appropriate histone pre-mRNA processing accumulation of aberrant mRNA isoforms with poly(A) tails occurs. Analysis of patients with biallelic mutations in LSM11 revealed an enrichment of misprocessed aberrantly polyadenylated transcripts and impairment of chromatin-bound histone stoichiometry. Thus, disrupting the nuclear distribution of cGAS. As discussed, nuclear-localized cGAS is tethered tightly to nucleosomes and prevented from detecting self-DNA. Patients’ fibroblasts displayed a constitutive ISG signature because of the constitutive production of cGAMP from soluble nuclear cGAS.

#### RNA sensor-mediated AGS

Adenosine deaminases acting on RNA (ADARs) are adenosine deaminases that convert adenosine to inosine in dsRNA via a process known as hydrolytic deamination (138). ADAR1 also plays a role in metabolizing basally transcribed retroelements to prevent the accumulation of dsRNA in the cell (139). ADAR1 has two isoforms, p150 and p110. p150 is located in the cytoplasm and is IFN inducible, whereas p110 is constitutively expressed in the nucleus (78). LOF ADAR1 mutations result in the development of AGS because of aberrant activation of the RNA sensor Mda5 and a constitutive IFN response. Conversion of adenosine to inosine by ADAR1 impairs the ability of Mda5 to bind dsRNA, thus preventing immune recognition of cellular RNA products (94). Mutations in the dsRNA binding regions of the catalytic domain of ADAR1 create protein instability and loss of deamination activity (16). Like other forms of AGS, patients with ADAR1-driven AGS display severe neurological symptoms including intracranial calcification, white matter disease, and severe developmental delay (16). Autosomal-dominant and autosomal-recessive mutations in *ADAR1* have also been identified in AGS patients suffering from bilateral striatal necrosis (BSN) and spastic paraplegia with all patients displaying an active ISG signature (95). Both patients with ADAR1 AGS mutations or ADAR1-deficient mice experience a severe type I interferonopathy because of cytoplasmic accumulation of dsRNA and activation of RNA sensors (16, 77). Development of mouse models of ADAR1 deficiency has provided overwhelming evidence for the important role ADAR1 plays in negatively regulating Mda5 activation (77, 78, 140, 141). Furthermore, the embryonic lethality of ADAR1-deficient mice is rescued by concurrent deletion with Mda5 or MAVS (78, 94). More recently, the precise signaling events downstream of Mda5 activation in *ADAR1* deficiency have been delineated using a mouse model of AGS. Mice expressing a single copy of the ADAR1 AGS allele P195A coupled to a p150 null allele (*Adar^P195A/p150^*^-^) displayed severe disease, which was dependent on Mda5, LGP2, type I IFNs, and PKR. Furthermore, pharmacological inhibition of the PKR-mediated integrated stress response (ISR) alleviated disease (77).

In addition to driving ADAR1-associated AGS, GOF mutations in the helicase domain of Mda5, encoded by *IFIH1*, have also been identified as an underlying cause of AGS (17, 73). As discussed, Mda5 is an important anti-viral sensor of dsRNA, which signals via the adaptor protein MAVS. GOF mutation in *IFIH1* results in enhanced RNA binding and an increase in the basal- and ligand-induced IFN responses. *IFIH1*-related AGS results in a constitutively active IFN response and ISG signature in patients. In addition to AGS *IFIH1,* SNP risk alleles have also been identified in a range of autoinflammatory and autoimmune conditions such as type 1 diabetes, vitiligo, psoriasis, arthritis, multiple sclerosis, and SLE (74, 96, 142–145).

### STING-associated vasculopathy with onset in infancy (SAVI)

GOF mutations in the gene encoding STING have been attributed to a rare interferonopathy known as STING-associated vasculopathy with onset in infancy (SAVI) (18, 146). Patients suffering from SAVI exhibit an elevated ISG expression signature in peripheral cells as well as respiratory failure, skin rash, and pulmonary fibrosis (147). An asparagine to serine mutation (N154S) has been identified as one of the most common mutations found in SAVI patients (148). However additional autosomal-dominant and autosomal-recessive mutations have been identified in the connector helix loop (N154S, V155M, and V147L) and the polymerization interface (G207E, R281Q, and R284G/R284S) (18, 102, 149). Considering these findings, studies using mouse models expressing SAVI mutations recapitulated human disease (103, 104, 150). Interestingly, heterozygous mice harboring one copy of the N153s allele display similar clinical symptoms to SAVI patients (103, 104). N153s mice also display defects in adaptive immune cell development, myeloid cell expansion, and a reduction of T cells in the thymus, blood, and spleen (104). Surprisingly, *Irf3* or *Ifnar* deficiency did not alleviate disease in N153S mice, suggesting that SAVI occurs independently of type I IFN (103, 104, 150). In contrast, the loss of TCRbeta T cells protects mice from disease (105, 150). In addition, N153S mice also develop chronic colitis in a microbiome-dependent manner (105). To date, there have been no published reports of colitis in SAVI patients. The severe interstitial lung disease, which presents in SAVI, is not collectively seen in all type I interferonopathies, indicating that the shared ISG signature can function as a diagnostic or danger signal for other interferon-independent mechanisms contributing to disease.

#### COPA syndrome

COPA is a subunit of the coatomer protein complex (COPI), which facilitates the retrograde transport of cargo proteins between the ER and Golgi and the localization of vesicles within the Golgi (151). A range of clinical symptoms in COPA syndrome have been reported, including a constitutive ISG signature, interstitial lung disease, renal disease, arthritis, and arthralgia. Myositis, macrophage activation syndrome, and autoantibodies have also been reported in patients with COPA syndrome (56). Interestingly, clinical penetrance can vary in COPA syndrome, including patients with an elevated ISG expression signature. Thus, additional environmental or genetic factors may influence the development of clinical disease (56). Given the clinical similarities with SAVI, it was hypothesized that COPA syndrome may be STING-dependent. Further analysis identified aberrant trafficking of STING from the ER to the Golgi as the underlying cause of COPA syndrome (48, 49, 56). Mutant COPA results in the accumulation of STING in the Golgi and constitutive activation of IFN signaling (49).

#### Stat1 gain-of-function mutations

STAT1 is a latent transcription factor activated downstream of type I, II, III IFNs, and IL-127 (79). STAT1 GOF mutations render patients highly susceptible to fungal infections because of decreased IL-17-producing T cells. Type I and I IFNs, which signal via STAT1, inhibit Th17 cells; thus, enhancement of IFN-induced STAT1 signaling has been attributed to impaired IL-17 production and susceptibility to *Candida* infection. Chronic mucocutaneous candidiasis (CMC) and hypothyroidism are common clinical manifestations of STAT1 GOF mutations (152). Other autoimmune symptoms include enterocolitis, immune cytopenia, endocrinopathies, and SLE (108). In comparison to other interferonopathies, STAT1 gain-of-function mutations result in high-level disease penetrance, with more than 65 recurrent GOF mutations in STAT1 identified in 400 patients globally (109). Mechanistically, STAT1 GOF mutations impair STAT1 dephosphorylation and create a locked conformation, which prevents nuclear to cytoplasmic shuttling (109). In addition, phosphorylated tyrosine 701 becomes inaccessible to phosphatases. Given the requirement of STAT1 in driving an ISG expression signature, the differential phenotypes of cGAS/STING-driven inflammatory conditions versus STAT1-driven disease may be attributed to IFN-independent pathways.

## UBIQUITIN ACCUMULATION IN INTERFERONOPATHIES

**Fig 3:**
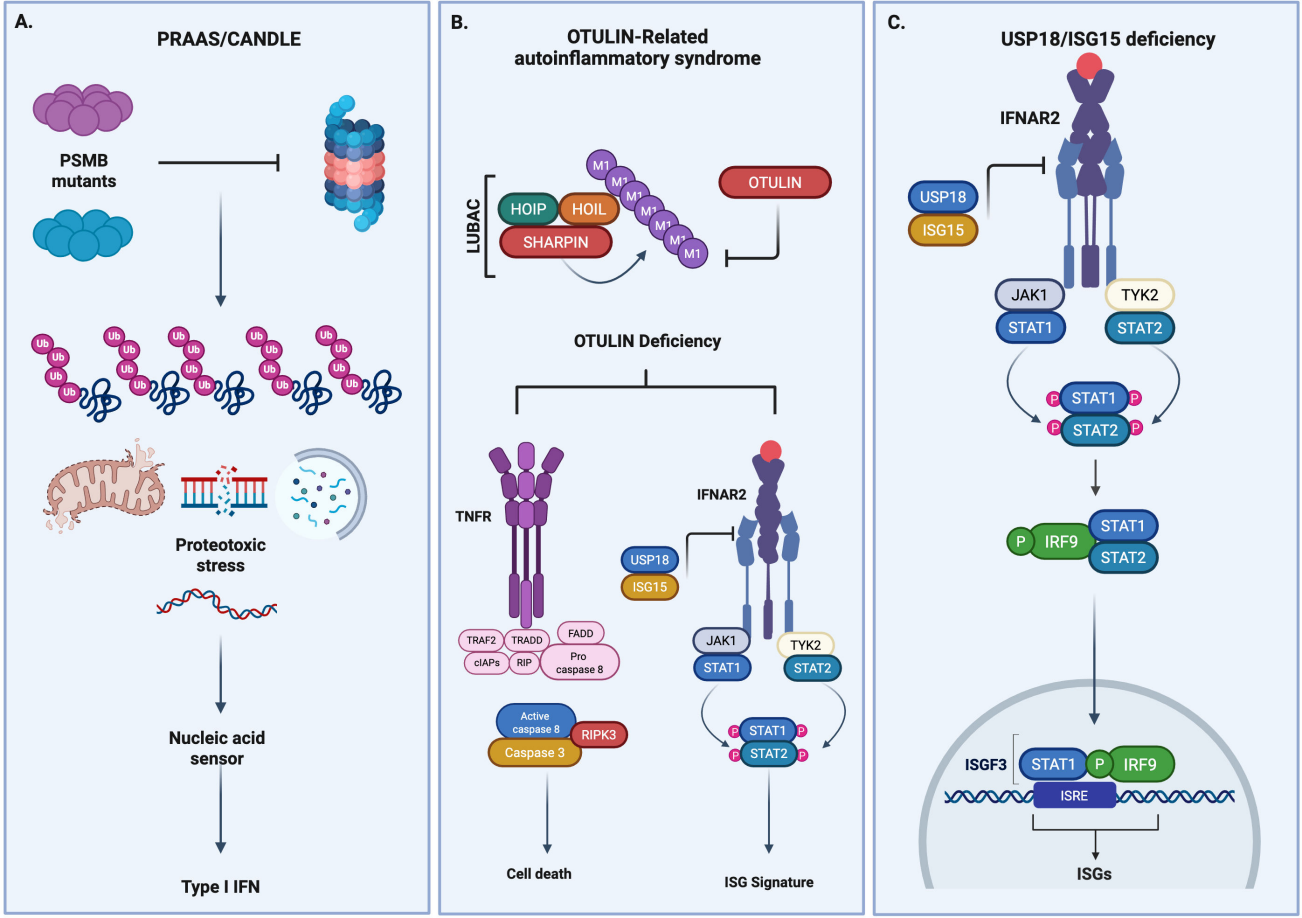
Ubiquitin accumulation promotes interferonopathies. Schematic representation of inborn error mutations that perturb ubiquitin and ubiquitin-like signaling molecules. (**A**) PSMB mutations disrupt the normal function of the proteasome. In the absence of a functioning proteasome, ubiquitinated proteins accumulate in the cell and promote proteotoxic stress and release of danger signals, which can trigger the activation of nucleic acid sensors and induction of an IFN response. (**B**) OTULIN counters LUBAC auto-ubiquitination, reduces LUBAC levels, and impairs the recruitment of LUBAC into complex I downstream of TNFR1. Impaired TNF-induced NFkB activation in OTULIN-deficient mice led to an increase in necroptosis, apoptosis, and embryonic lethality. Furthermore, in the absence of OTULIN, Caspase 8, and RIPK3, RIPK1 induced a robust type I IFN and ISG signature. (**C**) IFNAR activation stimulates the expression of USP18 and ISG15 as a negative feedback loop to limit IFNAR signaling. USP18/ISG15 deficiency promotes constitutive sustained activation of the IFNAR signaling pathway via enhanced interaction of JAK1 with STAT1 and promotes constitutive ISG expression.

Cytosolic accumulation of ubiquitin chains has been attributed to a range of conditions associated with excessive type I IFN and an ISG molecular signature as a result of proteotoxic stress (115, 116). Dysregulated or damaged mitochondria have been associated with ubiquitin labeling and mtDNA release to the cytosol in neurodegenerative diseases such as Parkinson’s; however, a growing number of interferonopathies also display evidence of ubiquitin accumulation and may represent an under-utilized danger signal to supplement genome sequencing-based diagnostics and ISG signatures. Inborn errors in the proteasome system and negative regulators of the TNFR1 and IFNAR pathways share underlying defects in eliminating ubiquitin chains and ubiquitin-like molecules (120). Ubiquitin chains serve as an anchor and scaffold between signaling complexes to sustain pathway activation and can contribute to the sustained activation of the type I IFN signaling pathway (71). In addition, the accumulation of ubiquitin on damaged proteins and organelles creates cellular stress and toxicity to trigger activation of innate immune signaling pathways and type I IFN production (115–117, 153) (Fig. 3). With respect to Mendelian inborn errors, numerous syndromes display constitutive ISG signatures because of the presence or absence of ubiquitin and ubiquitin-like molecules.

### ISG15/USP18 deficiency

Interferon-stimulated gene 15 (ISG15) is a member of the ubiquitin family of proteins and plays important functions in limiting viral replication and also regulates the IFNAR signaling pathway (154). Expression of ISG15 and the ISG15 conjugation machinery is induced by type I IFNs. Conjugation of ISG15 to host and viral proteins plays important roles in limiting viral replication and IFNAR signaling (5). Pro-ISG15 is expressed as a 17 kDa precursor and proteolytically cleaved into an active 15 kDa ubiquitin-like protein. ISG15 is covalently ligated to a target protein by a process known as ISGylation. ISG15 is a broad anti-viral effector protein. Indeed, *Isg15*-deficient mice are viable but are highly susceptible to a number of viral infections (155). Additionally, viruses can subvert ISGylation through the expression of ISG15 deconjugases to promote viral replication. Monogenic mutations in ISG15 have been identified in patients with symptoms including mycobacterial disease, dermatological disease, and an interferonopathy akin to AGS (8, 111, 112). The predominant AGS-like phenotype and interferonopathy have been attributed to constitutive IFNAR signaling. Following activation of the IFNAR pathway, IRF9 induces the expression of the ubiquitin-specific peptidase USP18 to bind IFNAR2 and sterically impair JAK1 binding. ISGylation of USP18 allows USP18 to stabilize and accumulate, thus impairing JAK1 signaling (156). In the absence of ISG15, USP18 is destabilized, resulting in constitutive activation of the IFNAR pathway. Despite the reported roles of ISG15 in suppressing viral replication no ISG15-deficient patients have presented with chronic viral infections.

Like ISG15 deficiency, USP18 deficiency leads to the development of a severe type I interferonopathy. USP18 deficiency results in the rapid onset of hydrocephalus, necrotizing cellulitis, systemic inflammation, and respiratory failure in the perinatal period (114). Homozygous mutation in USP18 results in the expression of a catalytically inactive form of USP18 (156). In the absence of USP18, sustained JAK-STAT signaling occurs and promotes the constitutive expression of an ISG signature. Remarkably, USP18-mediated inhibition of JAK1 occurs in a protease-independent manner (113, 156). Interestingly, type III IFN signaling is not regulated by USP18, and IFNλ signaling is sustained kinetically in comparison to IFNAR1/2 signaling.

### CANDLE/PRAAS

Proteasomes are complex structures that play critical roles in maintaining cellular homeostasis via the degradation of non-lysosomal, K48-ubiquinated proteins. IFNγ induced expression of β-subunits results in the formation of the immunoproteasome. β-subunits of the immunoproteasome consist of *PSMB9*, PSMB10, and *PSMB8* (157). The immunoproteasome plays an essential role in coping with the metabolic demands of an immune response. Loss of proteasome function results in the accumulation of polyubiquitinated proteins, cellular stress, and constitutive expression of type I IFNs (158). Recently, mutations in the immunoproteasomes have been identified as the underlying cause of a severe inflammatory disorder termed chronic atypical neutrophilic dermatosis with lipodystrophy and elevated temperature (CANDLE), a class of proteasome-associated autoinflammatory syndrome (PRAAS) (115, 118, 119). CANDLE, although triggered by numerous mutations, is characterized by the sustained production of type I IFN, which is a key feature of each phenotype. Periodic flares of CANDLE have been attributed to events such as cold stress and viral infections. Interestingly, skin lesions are a common feature of CANDLE and range from acral periotic lesions, to annular plaques, and periocular edema. Neurological defects are not common in CANDLE patients; however, encephalitis and basal ganglia calcifications have been identified in some groups of patients (115, 117). Lipodystrophic fat loss is also a key manifestation of CANDLE (116, 159).

A missense mutation in PSMB8, G197E, results in the reduced expression of PSMB8, elevated IL-6 production, and lipodystrophy through disturbed adipocyte differentiation, high-level neutrophilia in the dermis, and accumulation of ubiquitinated proteins in PSMB8 mutated cells (157, 158). In another study, patient mutations in the proteasome genes *PSMA3*, *PSMB*4, *PSMB9,* and *POMP* resulted in proteasome dysfunction and constitutive induction of type I IFN and an associated ISG signature. CANDLE-associated mutations specifically exhibit constitutive upregulation of IFN-α and IFN-β but not IFNλ. Additionally, cellular analysis of patient PMBCs identified NK cells and pDCs as the major source of type I IFN in CANDLE (118). Anti-TNF-directed therapies have proved ineffective at treating CANDLE (115). More recently, CANDLE patients treated with JAK inhibitors showed significant clinical improvement (106, 119). *Psmb8* knockout mice fail to recapitulate CANDLE phenotypes; thus, the precise molecular mechanisms that underpin CANDLE remain underexplored (160). Indeed, it is unclear which precise nucleic acid sensors dysfunctional proteasomes trigger to induce the IFN production and ISG expression observed in CANDLE patients. It has been proposed that the accumulation of defective ribosomal products and polyubiquitinated proteins may trigger a proteotoxic response through a nucleic acid sensor that leads to type IFN production (11). However, proteasome inhibition *in vitro* retains constitutive ISG activation in STING-deficient cells, suggesting that CANDLE occurs independently of cGAS and STING (118). Furthermore, cGAMP has not been detected in CANDLE patients.

### OTULIN-related autoinflammatory syndrome (ORAS)

OTULIN is a deubiquitinating enzyme that specifically hydrolyzes linear polyubiquitin chains (161). Hydrolysis of linear ubiquitin chains by OTULIN is essential for regulating downstream signaling of TNFR1 and IFNAR (158, 162). More recently, a reanalysis of linear ubiquitin signaling revealed that OTULIN counters LUBAC auto-ubiquitination, reduces LUBAC levels, and impairs the recruitment of LUBAC into complex I downstream of TNFR1 (163). Impaired TNF-induced NFκB activation in OTULIN-deficient mice led to an increase in necroptosis, apoptosis, and embryonic lethality. Furthermore, in the absence of OTULIN, Caspase 8, and RIPK3, RIPK1 induced a robust type I IFN and ISG signature (163). The severe inflammatory phenotypes identified in mouse models of OTULIN deficiency have also been recapitulated in human genetic inborn errors. To date, three missense or frameshift LOF mutations have been identified in ORAS. Mechanistically, ORAS mutations result in the loss of OTULIN deubiquitinating activity and reduced stability. In the absence of OTULIN, linear ubiquitin chains accumulate, which results in increased proinflammatory cytokine and neutrophilic dermatitis, panniculitis, and developmental delay. Liver disease is also recurrent in ORAS patients (164). ORAS patients also display fat loss and skin lesions, which are common features of CANDLE (165). Elevated levels of IL-18 and IFNγ have been routinely detected in patient serum. In addition, elevated expression of the ISGs IRF7, IFIT2, and Mx1 were also detected in whole blood and fibroblasts from patients with ORAS mutations. In another study, a homozygous missense, hypomorphic mutation in OTULIN was identified as the cause of an autoinflammatory condition. The resulting L272P mutation impaired ubiquitin binding and loss of stability. However, ISG expression was not assessed (121). Despite the recurrent detection of IFNγ and ISGs in ORAS patients, it remains unclear what role interferons play in the pathogenesis of ORAS.

## TYPE I IFN DEFICIENCY AND SUSCEPTIBILITY TO VIRAL INFECTION

Many viral infections range from asymptomatic to mild disease with self-limiting symptoms. However, severe viral infections can lead to hospitalization and death. Of note, respiratory infections account for 21% of all childhood mortality (166, 167). Type I and type III IFNs play critical roles in anti-viral immunity, and control of viral infection, and impaired IFN responses are commonly associated with susceptibility to viral infection. In recent years, human immunodeficiencies affecting IFN production and IFN responses have been identified in children with severe viral infections (168). Based on numerous studies, a hierarchy of clinical manifestations has emerged with respect to the pathway location of each LOF mutation. In addition, through genetic analysis of these patients, the essential roles and redundancy of receptors, adaptors, and transcription factors have been identified for immunity to specific human viruses.

### Herpes simplex encephalitis in IFN deficiencies

**Fig 4:**
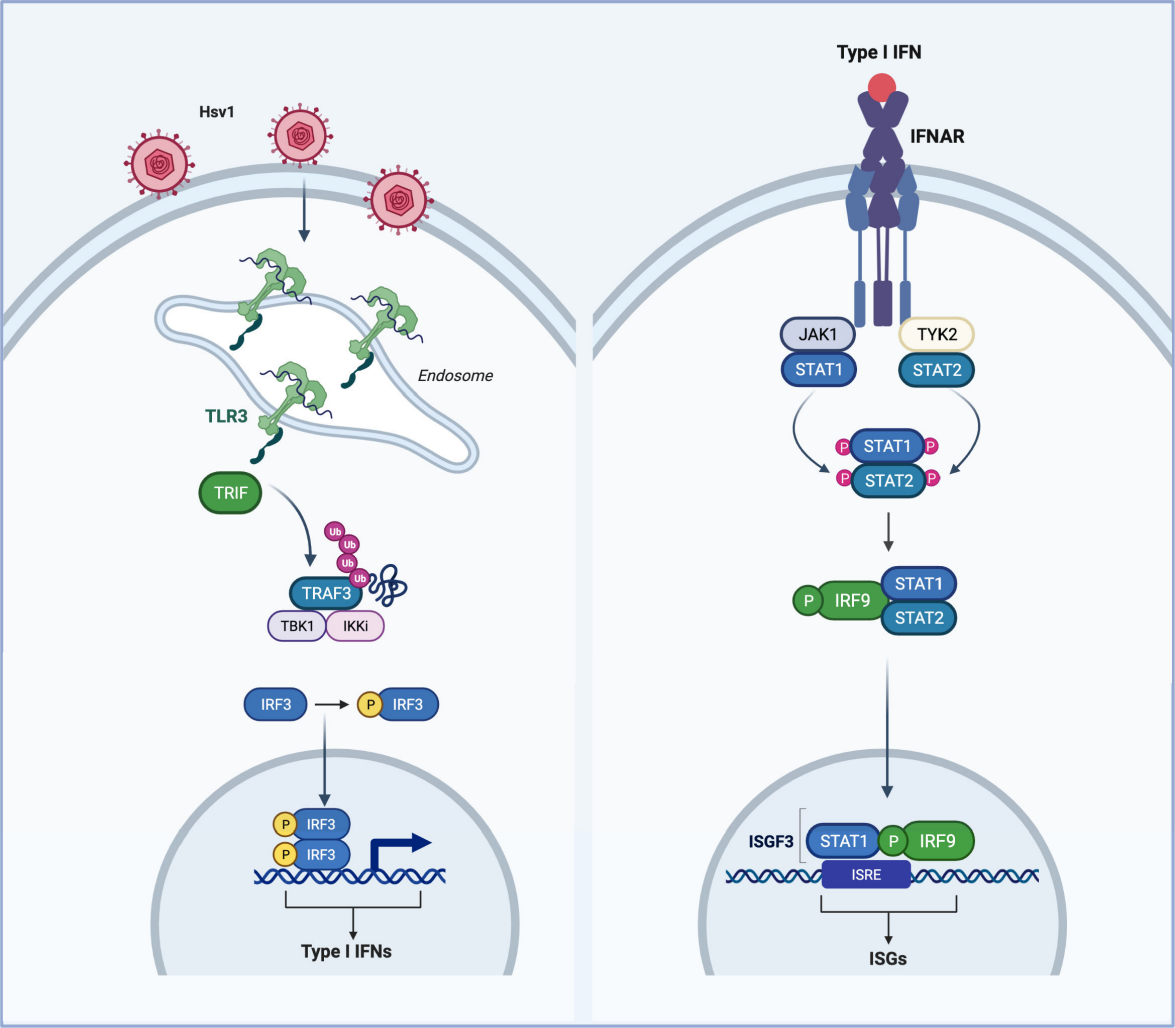
TLR3 signaling is essential for HSV1 immunity. Schematic representation of the TLR3 signaling pathway required for HSV1 immunity. Detection of viral RNA by TLR3 results in the recruitment of TRIF, TRAF3 ubiquitination, and recruitment of TBK1 and IKK. TBK1 and IKKε phosphorylate IRF3, which translocates and induces expression of IFN-β. IFN-β then induces the expression of anti-viral ISGs required for anti-HSV1 immunity. Loss-of-function mutations in TLR3, TRIF, TRAF3, TBK1, and IRF3 all lead to HSV1 susceptibility.

Children with germline mutations in components of the TLR-3 pathway are highly susceptible to herpes simplex encephalitis (HSE) due to impaired IFN responses (30, 35–37, 168, 169). HSV1 is a double-stranded DNA virus recognized by TLR-3 (170). TLR-3 plays a redundant role in responding to most microbes but is indispensable for natural immunity against HSV1 in the brain (Fig. 4). TLR-3 is expressed in the CNS in addition to dendritic and epithelial cells. HSV1 infects epithelial cells in the oral and nasal mucosa and permeates to the CNS via the trigeminal ganglia and olfactory nerves (171). HSE is a rare complication of HSV1 infection and presents in children with single-gene inborn errors of the TLR-3 pathway, including *TLR-3, TRIF, UNC93B1, TRAF3, TBK1,* and *IRF3* (30, 35–37, 124, 168).

Dominant negative mutations in *TLR-3* have been identified in children with severe forms of HSE (35). Typically, patients present with bilateral hemorrhagic lesions and a positive HSV1 PCR test. TLR-3-deficient patient fibroblasts and MDDCs also display impaired TLR3 responses to Poly (I:C). HSE has also been attributed to autosomal-recessive (AR) and autosomal-dominant (AD) TRIF deficiency (37). AR and AD mutations in TRIF resulted in loss of protein expression or the expression of a non-functioning protein, respectively. Interestingly, in cases of TRIF deficiency, relatives with TRIF deficiency positive for HSV1 infection did not present with HSE, indicating incomplete disease penetrance (37). UNC93B1 is an essential mediator of endosomal TLR signaling. AR UNC93B1 deficiency can also manifest as HSE as a result of impaired endosomal TLR responses (30).

In addition to TLR-3 and TRIF, AD mutations in *TRAF3* have also been identified in children with HSE. *TRAF3* mutant alleles were associated with loss of function-dominant negative forms of TRAF3 with impaired but not abolished TRAF3-dependent TLR-3 responses (36). Given that TRAF3-deficient mice die in the neonatal period, there lack definitive experimental data on the physiological functions of TRAF3 with respect to viral infection. TLR-3-independent pathways can compensate for the loss of TLR3 to restrict HSV1 replication. Indeed, activation of cGAS STING in microglia is essential for HSV1 immunity and production of IFN. Indeed, it has been proposed that IFN produced downstream of cGAS/STING promotes the upregulation of TLR-3 to potentiate anti-HSV1 immunity (171). However, there may exist non-redundant functions for TLR-3 in other cell types and tissues infected with HSV1. Based on these studies, converging PRR pathways require IFN and IFNAR signaling, given the severity associated with inborn errors in the TLR-3 pathway. Despite strong evidence supporting a role for cGAS and STING in anti-HSV1 immunity in experimental murine models, the current genetic evidence supports an indispensable role for TLR-3 signaling in controlling HSV1 infection in the CNS (7, 171, 172). Additionally, to date, the LOF STING haplotype, HAQ, which impairs STING responses, has not been attributed to susceptibility to HSV1 infection (173).

As discussed previously, TBK1 is an essential mediator of IRF3-induced type I IFN and inflammatory cytokine production. LOF mutations in *TBK1* have further identified a requirement for TLR-3 signaling and TBK1-mediated IFN production in controlling HSV1 infection in the CNS. Indeed, loss-of-function mutations in TBK1 have been identified in patients with heterozygous mutations D50A and G159A, resulting in protein instability and loss of kinase function, respectively (124). In these cases, elevated viral replication and cell death responses to HSV1 and VSV were observed in patient fibroblasts due to loss of IFN. More recently, TBK1 function has extended beyond IFN production and is identified as an important mediator of autophagy and suppression of RIPK1-dependent TNFR1-induced cell death (174–177).

### RNA virus immune deficiencies

Considering the SARS-CoV2 pandemic, many genetic studies have identified the type I IFN response as an essential mediator of SARS-Cov2 immunity and prevention of severe disease (122). Indeed, patients with neutralizing autoantibodies targeting IFN-alpha2 IFN were present in 10% of severe COVID-19 cases. In addition, several patients with severe forms of SARS-CoV2 infections harbored autosomal-recessive deficiencies in *IRF7* and IFNAR or autosomal-dominant deficiencies in *TLR-3, UNC93B1, TRIF, TBK1, IRF3, IRF7*, *IFNAR1,* and *IFNAR2* (122). Inborn errors in the TLR-3 pathway and IRF7-dependent type I response were causative factors in a percentage of severe COVID-19 cases. In addition to SARS-CoV2 mutations in the transcription factor, IRF7 has been identified as a risk allele for severe H1N1 influenza infection. IRF7 is an ISG induced to amplify type I and type III IFN and plays an important role in pDCs. The RNA-sensor Mda5 (*IFIH1*) is an essential mediator of type I IFN production following infection with Rhinovirus or respiratory syncytial virus (RSV). In one study, 120 otherwise healthy children with severe respiratory viral infections were sequenced. Loss-of-function variants in *IFIH1* encoding Mda5 were identified as the cause of elevated viral replication. Mda5 mutations impaired IFNB as a result of reduced stability and loss of ATPase activity (75). In addition, another study identified homozygous missense mutations in Mda5 that failed to recognize Poly IC. Increased viral replication was observed in the nasal epithelial cells. Thus, Mda5 deficiencies result in impaired sensing and IFN responses leading to enhanced susceptibility to common infections such as IAV, RSV, and rhinovirus (178).

## FUTURE OUTLOOK

The pathogenic role of IFNs in disease has been a well-established feature of many inflammatory diseases. More recently, elucidation of the key signaling pathways that precede IFN production coupled with the availability of exome sequencing for diagnostic use has revealed, at a molecular level, the causal factors that perturb innate immune signaling pathways and initiate the development of interferonopathies. Despite the broad detection of ISG signatures as an indicator of interferonopathy, the preceding pathways that are triggered are more complex and contribute to disease independently of IFN. Indeed, ISG signatures in inborn errors outside the IFNAR pathway represent a secondary response to the primary perturbed pathway. In addition, type I, II, and III interferon will activate comparable ISG signatures despite originating from diverse cell types and signaling pathways. Upregulation of USP18 represents a definitive marker of type I IFN dysregulation is not upregulated by type III IFN. Thus, its inclusion in diagnostic ISG panels may indicate more specificity in patient tissue analysis. Although the ISG signature offers a very valuable diagnostic tool, a wider analysis of the pathway-specific factors would offer significant value for early diagnosis. For example, the differentiation between diseases triggered by RNA sensors versus DNA sensors often requires a significant amount of molecular elucidation prior to defining the mechanistic basis of a novel interferonopathy. More routine quantification of cGAMP levels and improved cGAMP detection methods would assist with defining diseases that promote constitutive activation of cGAS and STING.

Current treatment options for interferonopathies rely on direct targeting of the IFNAR receptor or JAK inhibition. Thus, any IFN-independent pathologies associated with the disease are not impacted. Unsurprisingly, therapies targeting type I IFN have failed to gain significant traction as a frontline therapy for interferonopathies. Furthermore, evidence is now emerging that certain interferonopathies are driven independently of IFN. Indeed, in experimental models of SAVI, lung and intestinal pathologies occur independently of type I IFN. This indicates that other pathways downstream of STING mediate interstitial lung disease associated with SAVI. Furthermore, STING-driven diseases such as SAVI and COPA syndrome uniquely display severe interstitial lung disease, typically, not seen in other conditions such as AGS. The differential manifestation of diseases that share excessive IFN is especially relevant to the success of how these diseases are treated in the future. As our knowledge of the underlying mechanisms of these diseases grows, there is now a huge focus on developing the next generation of therapies to directly target key proteins that mediate interferonopathies such as nucleic acid sensors like cGAS, STING, and Mda5.

Despite the essential roles of STING in mediating protective immunity against DNA viruses in experimental models, very little evidence exists for anti-viral STING functions in human systems. Therefore, direct targeting of STING may not render patients vulnerable to viral infections as seen with anti-IFNAR therapies. As discussed, TLR3 signaling is an essential component of immunity to HSV1 infection and protection of HSE. The HAQ STING allele is a LOF function variant that impairs the induction of type I IFN. Despite being the second most common allele, the HAQ variant does not affect HSE susceptibility. It is unclear if the HAQ allele impacts disease penetrance in cGAS/STING-driven interferonopathies. To date, no LOF cGAS mutations have been identified. Viral tropism and infectivity of specific cell types where cGAS and STING may not be expressed might also contribute to cGAMP redundancy in anti-viral immunity. Furthermore, DNA viruses do not typically spread as efficiently as RNA respiratory viruses. Thus, in a viral context, the physiological relevance of cGAMP may be limited to host-derived DNA.
